# Effect of radiotherapy on the surface roughness and microhardness of contemporary bioactive restorative materials

**DOI:** 10.1007/s00520-024-08476-5

**Published:** 2024-04-18

**Authors:** Cansu Atalay, A. Ruya Yazici

**Affiliations:** Department of Restorative Dentistry, School of Dentistry, Hacettepe University, Ankara, Turkey 06230

**Keywords:** Radiotherapy, Surface roughness, Vickers hardness, Bioactive materials

## Abstract

**Objective:**

The aim of this in vitro study was to evaluate the effect of radiotherapy on the surface microhardness and roughness of different bioactive restorative materials.

**Materials and methods:**

A total of 60-disc specimens (5 mm × 2 mm) were performed in four groups (*n* = 15 each) from Equia Forte HT, Cention N, Activa Bioactive Restorative, and Beautifil II. Following the polishing procedure (600, 1000, 1200 grit silicon carbide papers), all specimens were irradiated at 2 Gy per fraction, five times a week for a total dose of 70 Gy in 30 fractions over 7 weeks. Before and after the irradiation, the specimens were analyzed regarding the surface roughness and microhardness. Surface morphology was also analyzed by scanning electron microscopy. Kruskal–Wallis test, Wilcoxon test, and paired sample *t*-test were used for statistical analysis.

**Results:**

Significant differences were found after radiation with increased mean roughness of both Cention N (*p* = 0.001) and Beautifil II (*p* < 0.001) groups. In terms of microhardness, only the Beautifil II group showed significant differences with decreased values after radiation. There were statistically significant differences among the groups’ roughness and microhardness data before and after radiotherapy (*p* < 0.05).

**Conclusion:**

The effect of radiotherapy might differ according to the type of the restorative material. Although results may differ for other tested materials, giomer tends to exhibit worse behaviour in terms of both surface roughness and microhardness.

**Clinical relevance:**

In patients undergoing head and neck radiotherapy, it should be taken into consideration that the treatment process may also have negative effects on the surface properties of anti-caries restorative materials.

## Introduction

Tooth caries ranks among the most prevalent chronic ailments affecting people worldwide. This condition can affect individuals throughout their lives. While the primary cause is the intricate interplay of factors like oral hygiene, nutrition, and saliva quality, there are also exceptional circumstances known as radiation caries. Radiotherapy stands as the predominant treatment method for malignant tumors located in the head and neck regions. This therapeutic approach employs high-energy X-rays at doses ranging from 40 to 70 Gy. Even low doses of radiation can significantly diminish a patient’s quality of life [1]. It leads to alterations not only in saliva production but also in its composition, rendering patients susceptible to caries due to hyposalivation. In regions where caries would not typically occur, such as the incisors of the anterior teeth, the tubercule areas of the posterior teeth, and the lingual and vestibule surfaces of the anterior and posterior teeth, the tooth structure becomes demineralized, resulting in radiation caries [2].

When planning restorative treatment, numerous factors must be taken into account, including the risk of caries, the patient’s overall health, patient cooperation, and, most importantly, the choice of restorative materials [3]. The protocol for dental restorations in oral cancer patients remains a subject of ongoing debate. Consequently, the selection of the most suitable restorative material for patients undergoing radiotherapy appears to rely on the clinical judgment of the healthcare professional. Given the heightened susceptibility to caries in radiotherapy patients, it would be prudent to opt for advanced restorative materials. Calcium, phosphate, and fluoride ions are the most commonly associated elements in enhancing the resilience of dental tissue against acid attacks [4, 5].

Glass ionomers, in particular, offer a broad spectrum of clinical applications due to their commendable bond strengths and biocompatible qualities that release fluoride [6]. Nevertheless, certain drawbacks, such as inadequate wear resistance, low flexural strength, and susceptibility to moisture during the initial setting stages, have restricted their usage [6]. Recent developments aimed at eliminating the disadvantages of glass ionomers have led to the introduction of Equia Forte HT (GC Corp.), a glass hybrid material enhanced with properties such as higher wear resistance and fluoride release [7, 8].

Cention N (Ivoclar-Vivadent) is an innovative tooth-colored restorative material known for its exceptional bending strength, which it owes to its cross-linked polymer structure. This material falls under the category of alkacids and is primarily composed of UDMA (Urethane Dimethacrylate), consisting of both a powder and a liquid component, offering the flexibility of self-curing or light-curing options. One of its noteworthy features lies in the alkaline glass particles incorporated into its composition. These particles play a crucial role in releasing fluoride, calcium, and hydroxyl ions when the material encounters acid attacks [9]. Importantly, these ions have a neutralizing effect on the pH level of the surrounding environment [10]. This pH-neutralizing action helps to prevent demineralization of the tooth structure and lays the foundation for the process of remineralization, thereby promoting overall dental health [11, 12].

A new RMGIC restorative called ACTIVA Bioactive Restorative (Pulpdent Corp.) has been recently introduced. According to the manufacturer, this material is described as a bioactive self-adhesive material that has the ability to stimulate hydroxyapatite formation and promote tooth remineralization. This is achieved through the release of calcium, phosphate, and fluoride [13]. By releasing these essential ions during its interaction with the tooth, ACTIVA aims to support the natural remineralization process, which can be beneficial for maintaining and strengthening tooth structure over time.

Another contemporary restorative material; Giomer (Beautifil II, Shofu) is a type of dental restorative material that combines the benefits of glass ionomer and composite resin materials [14]. It is a bioactive material that releases and recharges fluoride, providing long-term protection against tooth decay. With its excellent aesthetic properties closely resembling natural tooth color and translucency, Giomer has gained popularity as a versatile and reliable option in modern restorative dentistry.

To the best of our knowledge, only few studies assessed the effect of radiotherapy on restorative materials’ physical and mechanical properties [15–18]. Furthermore, with the introduction of bioactive restorative materials, it is paramount to investigate its’ effect on these restoratives as they are also advocated in patients who needs radiotherapy treatment. Therefore, the aim of this in vitro study was to evaluate the effects of radiotherapy on surface microhardness and roughness of contemporary bioactive restorative materials. The tested null hypothesis tested were the following: (1) Radiotherapy does not influence the surface microhardness of tested materials, (2) there would be no difference in surface roughness of tested materials after radiotherapy.

## Materials and methods

### Study materials

Four groups were created according to the tested bioactive restorative materials. The study materials were as follows: Equia Forte HT (GC Corp., Tokyo, Japan), Cention N (Ivoclar-Vivadent AG, Schaan, Liechtenstein), Activa BioActive Restorative (Pulpdent Corp., Watertown, MA, USA), and Beautifil II (Shofu, San Marcos, CA, USA). The composition of each tested material can be found in Table 1. For the statistical analysis, G*Power software (Ver 3.1, Heinrich-Heine Dusseldorf University, Dusseldorf, Germany) was employed. The analysis was conducted using a one-way ANOVA-type power analysis with a 95% confidence interval, 80% power, and 0.50 effect size values. Based on the sample size calculation, it was determined that approximately 12 specimens were needed for each group in order to detect a difference of 25% among the study groups. Therefore, a total of 15 specimens per group were used to enhance the statistical power of the study.

**Table 1:** Descriptions of the materials used in the study

Material—material type	Manufacturer/lot no	Composition
Equia Forte HT—*Bulk-fill glass hybrid*	GC Corp., Tokyo, Japan/1905231	Powder: fluoroaluminosilicate glass, polyacrylic acid, iron oxide Liquid: polybasic carboxylic acid, water
Cention N—*non-adhesive bulk-fill resinous material*	Ivoclar-Vivadent, Schaan, Liechtenstein/Z03CKR	Powder: barium aluminum silicate glass, ytterbium trifluoride, isofiller, calcium barium, aluminum fluorosilicate glass, calcium fluoro, silicate glass Liquid: urethane dimethacrylate, tricyclodecandimethanol dimethacrylate, tetramethyl-xylylene diurethane dimethacrylate, polyethylene glycol 400 dimethacrylate, ivocerin, hydroxyperoxide
Activa BioActive Restorative—*self-adhesive bulk-fill resinous material*	Pulpdent, Watertown, MA, USA/220329	Powder: silanated bioactive glass and calcium, silanated silica, sodium fluoride Liquid: diurethane modified by the insertion of a hydrogenated polybutadiene and other methacrylate monomers, modified polyacrylic acid
Beautifil II—*Giomer*	Shofu Dental, Kyoto, Japan/031917	Bis-GMA 7.5%, triethylenglycol dimethacrylate 5%, aluminofluoro-borosilicate glass 7.5%, Al2O3, DL-camphorquinone

### Preparation of specimens

A customized cylindrical teflon mold with internal dimensions of 5 mm in diameter and 2 mm in height was used to prepare the specimens. The top and bottom of each specimen were covered with a Mylar strip (S.S. White Limited, Middx, UK) and glass slide, and the top surface was condensed with constant finger pressure. Each restorative material was prepared as described in the material’s manuals:

#### Equia Forte HT

The Equia Forte HT capsule was activated and mixed using an automixer (Softly, Satelec Acteon, Merignac Cedex, France). The resulting mixture was promptly inserted into the mold. Once the setting process was finished, Equia Forte Coat was applied using a micro-tip applicator without air blowing. Subsequently, it was light-cured for 20 s using a LED curing unit (440–480 nm, 1500 mW/cm^2^, Radii plus, SDI, Victoria, Australia).

#### Cention N

For Cention N, a standard powder-to-liquid ratio of 4.6:1 was used, meaning one scoop of powder was mixed with one drop of liquid. The powder and liquid components were dispensed onto a mixing pad and then mixed together using a plastic spatula. The mixing process followed the manufacturer’s instructions and lasted for 45 to 60 s. Once properly mixed, the material was inserted into the mold as per the experimental procedure.

#### Activa BioActive Restorative

Activa Bioactive was injected in excess inside the mold and light-cured for 20 s using a LED curing unit.

#### Beautifil II

The giomer restorative material was inserted into the mold using a flat-surface hand instrument. After placement, it was light-cured for a duration of 20 s using a LED curing unit.

After the specimens were removed from the molds, they underwent a polishing process using a polishing machine (Presi Mecapol 220, Eybens, France). Silicon carbide papers with grit sizes of 600, 1000, and 1200 (Interflex, Ankara, Turkiye) were used for polishing. Each sandpaper was applied to the specimens under a 220 g axial load with water-cooling for a duration of 20 s. Following the polishing step, the specimens were subjected to ultrasonic cleaning using a device (Amsco, Reliance Sonic 250, Steris Corp., Mentor, OH, USA) with distilled water. The ultrasonic cleaning process lasted for 5 min to ensure thorough cleaning of the specimens.

### Radiotherapy procedure

The specimens underwent irradiation at a dose of 2 Gy per fraction, administered five times a week. This treatment regimen was carried out over a period of 7 weeks, resulting in a total dose of 70 Gy delivered in 30 fractions. The radiotherapy procedure was conducted in the Hacettepe University Oncology Hospital setting using a linear accelerator (Elekta Versa HD, Stockholm, Sweden).

### Microhardness tests

The surface microhardness values were determined using a digital microhardness tester (Shimadzu HMV-G, Shimadzu Corp., Japan). To obtain these values, three indentations were made on each specimen, ensuring that they were at least 1 mm apart from each other. A 100 g load was applied for a duration of 15 s during the indentation process. The Vickers hardness number (VHN) was calculated by averaging the three values obtained from the indentations.

### Surface roughness tests

The mean surface roughness (Ra) values for all specimens were measured with a profilometer (Perthometer M1, Mahr GmbH, Go¨ttingen, Germany). Three successive measurements were taken in different directions for each surface, and the average surface roughness values were recorded. The cutoff value for surface roughness was set at 0.25 mm, and the sampling length for each measurement was 1.5 mm. Prior to each measurement session, the profilometer was calibrated to ensure accurate results.

### Scanning electron microscopy (SEM) analysis

For surface morphology analysis, one specimen was randomly chosen from each group. These selected specimens underwent a gold sputtering process and were then examined using scanning electron microscopy (SEM) (Nova NanoSEM 430, FEI, Hillsboro, OR, USA) at 100 × and 1.00 k × magnifications.

### Statistical analysis

The statistical analysis was carried out using SPSS software (Version 21.0, SPSS Inc., Chicago, IL, USA). The data were subjected to the Shapiro–Wilk normality test and were shown to follow a non-normal distribution. The Kruskal–Wallis test was used for the materials’ hardness and roughness data comparison before and after radiotherapy. For comparisons of radiotherapy effects within each restorative material’s hardness test, the Wilcoxon test was used for the Activa group and the paired sample *t*-test was used for the rest of the tested restorative materials. For roughness data comparison, the Wilcoxon test was used for the Cention N group, and the paired sample *t*-test was used for the rest of all groups (*p* < 0.05).

## Results

There were statistically significant differences among the groups’ roughness data before and after radiotherapy (*p* < 0.05).

The Beautifil II group showed statistically significantly lower roughness values than other materials before radiotherapy (*p* < 0.05) (Table 2). While no differences were detected between Activa and Equia Forte HT after radiotherapy (*p* = 0.151), statistically significant differences were found between the other tested groups (*p* < 0,05). While radiotherapy caused a statistically significant increase in the roughness of Cention N (*p* = 0.001) and Beautifil II groups roughness data (*p* < 0.001), respectively, no difference was found within the other tested materials (*p* > 0.05).

**Table 2:** Mean surface roughness and standard deviations for each restorative material before and after radiotherapy (*n* = 15)

Groups	Mean Ra ± SD		
	Before radiotherapy	After radiotherapy	*p*
Equia Forte HT	0.292 ± 0.113^a^	0.361 ± 0.132^a^	*0.173*
Cention N	0.289 ± 0.097^a^	0.676 ± 0.236^b^	*0.001*
Activa BioActive Restorative	0.234 ± 0.053^a^	0.264 ± 0.046^a^	*0.140*
Beautifil II	0.117 ± 0.44^b^	0.184 ± 0.208^c^	*0.00*

Means followed by same superscript letter within the same column are not statistically different (*p* > 0.001)

In terms of surface microhardness, statistically significant differences were found between materials before and after radiotherapy (Table 3). Before radiotherapy, Beautifil II groups’ microhardness data were found to be significantly higher than the other materials tested (*p* < 0.05). After radiotherapy, the Equia Forte HT group showed significantly lower values than the other restorative materials (*p* < 0.05).

**Table 3:** Mean surface microhardness and standard deviations for each restorative material before and after radiotherapy (*n* = 15)

Groups	Mean Ra ± SD		
	Before radiotherapy	After radiotherapy	*p*
Equia Forte HT	54.696 ± 12.356^a^	50.603 ± 0.403^a^	*0.306*
Cention N	67.774 ± 12.743^a^	67.529 ± 13.590^b^	*0.966*
Activa BioActive Restorative	65.665 ± 13.033^a^	79.126 ± 64.377^b^	*0.334*
Beautifil II	81.308 ± 8.349^b^	74.632 ± 5.085^b^	*0.026*

Means followed by the same superscript letter within the same column are not statistically different (*p* > 0.001)

When comparing the radiotherapy effect within each group, a statistically significant difference was only detected in the Beautifil II groups’ surface hardness data (*p* = 0.026). The radiotherapy reduced the surface hardness of the Beautifil II group. In terms of roughness, radiotherapy increased Cention N (*p* = 0.001) and the Beautiful II group’s roughness (*p* < 0.001).

### SEM analysis

**Fig. 1:**
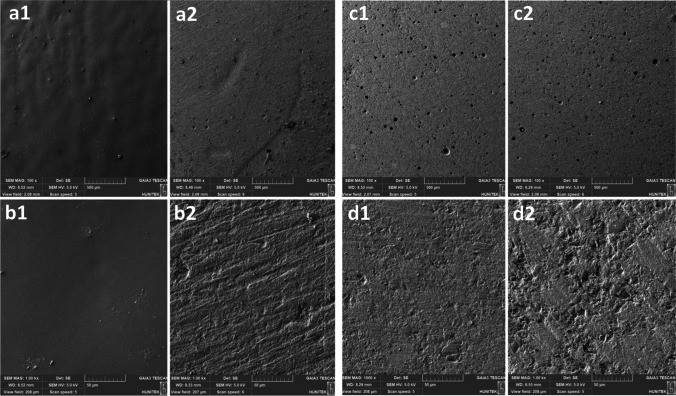
Scanning electron microscope images (a1) Equia Forte HT specimens at 100 × magnification before radiotherapy; (a2) Equia Forte HT specimens at 100 × magnification after radiotherapy; (b1) Equia Forte HT specimens at 1.00 k × magnification before radiotherapy; (b2) Equia Forte HT specimens at 1.00 k × magnification after radiotherapy; (c1) Cention N specimens at 100 × magnification before radiotherapy; (c2) Cention N specimens at 100 × magnification after radiotherapy; (d1) Cention N specimens at 1.00 k × magnification before radiotherapy; (d2) Cention N specimens at 1.00 k × magnification after radiotherapy

**Fig. 2:**
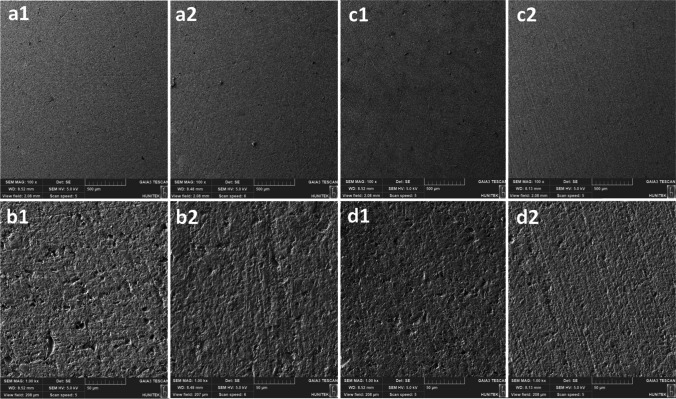
Scanning electron microscope images (a1) Activa Bioactive specimens at 100 × magnification before radiotherapy; (a2) Activa Bioactive specimens at 100 × magnification after radiotherapy; (b1) Activa Bioactive specimens at 1.00 k × magnification before radiotherapy; (b2) Activa Bioactive specimens at 1.00 k × magnification after radiotherapy; (c1) Beautifil II specimens at 100 × magnification before radiotherapy; (c2) Beautifil II specimens at 100 × magnification after radiotherapy; (d1) Beautifil II specimens at 1.00 k × magnification before radiotherapy; (d2) Beautifil II specimens at 1.00 k × magnification after radiotherapy

Based on the scanning electron microscopy images, the topographical changes became more pronounced in all groups after undergoing radiotherapy (Figs. 1 and 2). These changes included the presence of irregular surfaces and superficial scratches, indicating some degree of degradation. However, it is important to note that there were limited distinct differences observed between the various groups in terms of these topographical changes.

## Discussion

In the present study, the effect of radiotherapy on surface hardness and roughness of newly introduced bioactive restoratives was investigated. As the surface hardness of Beautifil II was decreased after radiotherapy exposure, the first hypothesis, that radiotherapy does not influence the surface hardness of tested materials, had to be partially accepted. de Amorim et al. [16] have investigated radiotherapy’s impact on various restorative dental materials’ surface properties. They observed that bulk-fill resin composite and resin-modified glass ionomer cement exhibited statistically similar hardness values before and after radiation exposure. In contrast, conventional resin composite showed an increase in hardness values, while conventional glass ionomer cement demonstrated a decrease in hardness values following radiotherapy. However, it is worth noting that a previously published study indicated that gamma radiation actually increased the microhardness of glass ionomer cements [19].

This result was also confirmed by another study that observed an increase in the microhardness of resin composite and a glass ionomer after ionizing radiation exposure [20]. In a study, the microhardness increase after radiotherapy was explained by the materials’ resin-rich layer damage caused by radiotherapy that induced the exposure of inorganic filler particles [16]. Unfortunately, there are conflicting results in the literature regarding the radiotherapy effects on restorative material.

In a most recent study, the effects of irradiation on the novel alkasite and glass hybrid materials’ mechanical, chemical, and surface properties were investigated [21]. It was concluded that radiotherapy has neither a detrimental nor a beneficial effect on the microhardness of alkasite, resin composite, and glass hybrid restorative material, which is consistent with our findings. In the present study, although not statistically significant, only the hardness of Activa was increased after radiotherapy exposure. However, the lack of information regarding the effects of radiotherapy on Activa BioActive Restorative made it difficult to compare our findings.

Brandeburski and Della Bona [22] evaluated the radiation effect on resin composites and conventional and resin-modified glass ionomer cements. In that study, one of the tested resin composites’ hardness was decreased, and glass ionomers’ roughness and hardness were increased. No significant effect of radiotherapy on the rest of the materials was observed. They have stated that the hardness of resin composites can be affected by radiotherapy in two ways, either by breaking the bonds within the polymer network which results in the reduced hardness or increased hardness due to the crosslinking enhancement of the polymer network. Another study also found the adverse effect of radiotherapy on the hardness of resin composites [23].

In our study, only the hardness of giomer material—Beautifil II—was found to be negatively affected by radiotherapy. No change was detected in surface hardness among the rest of the tested materials which were bulk-fill glass hybrid, alkasite, and self-adhesive bulk-fill resinous material. This could be related to the composition of the material which is more similar to the conventional resin composite. The polymer network in giomer might have been broken with the influence of radiotherapy exposure.

Regardless of radiation, this might also be explained by the water sorption of the material. The resin matrix of Beautifil II could have been softened through water absorption, as it does not contain hydrophobic monomer, urethane dimethacrylate (UDMA). This structure could lead to the hydrolytic breakdown of the bond between the filler particles and resin matrix, resulting in reduced hardness [24].

Debonding of the fillers in the material through water absorption could also be an explanation for the increased roughness values observed in the Beautifil II group. In terms of surface roughness, only the Beautifil II and Cention N groups were found to be negatively affected by the radiotherapy. The rest of the tested material’s roughness was also increased, but this was statistically insignificant. Therefore, the second hypothesis that there would be no difference in surface roughness of tested materials after radiotherapy should be rejected partially. This finding might be related to the partially reacted or unreacted calcium glass fillers used in the composition of giomer and alkasite.

In another study, radiotherapy increased the roughness values of the glass ionomer materials which is opposing to our results [17]. However, in that study, the tested glass ionomers were Ketac Molar Easymix, Vitro Molar, and Vitremer, which were different from the glass hybrid ionomer, Equia Forte HT, used in our study. Lima et al. explained their findings by the way how radiotherapy interacts with the water-based cements that form oxygen-reactive materials [17].

There are several limitations in the present study. First, unlike a clinical environment, the specimen was stored in the distilled water. Besides, the measurements were performed immediately after the radiotherapy exposure. Extended storage time in artificial saliva might have overcome the deleterious effect on the surface characteristics of the material. Moreover, the findings obtained in this study cannot be extrapolated to other materials as the compositional differences might probably affect the outcomes and generate different results. Unfortunately, there are conflicting results in the literature regarding the radiotherapy effects on restorative material. It is still uncertain whether irradiation affects them or not. Therefore, future research may need to evaluate other mechanical properties in clinical settings.

## Conclusion

Radiotherapy had no deleterious effect on the microhardness of tested bioactive restorative materials except for giomer, Beautifil II, where lower microhardness values were observed. Only the Beautifil II and Cention N group’s roughness values were found to be negatively affected by the radiotherapy while the roughness of the rest of the tested bioactive restorative materials was not altered. The effect of radiotherapy might differ according to the type of the restorative material.

## Data Availability

No datasets were generated or analyzed during the current study.
